# Fragment-based virtual screening identifies novel leads against Plasmepsin IX (PlmIX) of *Plasmodium falciparum*: Homology modeling, molecular docking, and simulation approaches

**DOI:** 10.3389/fphar.2024.1387629

**Published:** 2024-05-23

**Authors:** Haider Thaer Abdulhameed Almuqdadi, Sumaiya Kifayat, Razique Anwer, Jihad Alrehaili, Mohammad Abid

**Affiliations:** ^1^ Medicinal Chemistry Laboratory, Department of Biosciences, Jamia Millia Islamia, New Delhi, India; ^2^ Department of Chemistry, College of Science, Al-Nahrain University, Baghdad, Iraq; ^3^ Department of Pathology, College of Medicine, Imam Mohammad Ibn Saud Islamic University (IMSIU), Riyadh, Saudi Arabia

**Keywords:** Plasmepsin IX, antimalarials, homology modeling, fragment-based drug design, molecular docking, molecular docking simulation

## Abstract

Despite continuous efforts to develop safer and efficient medications, malaria remains a major threat posing great challenges for new drug discovery. The emerging drug resistance, increased toxicities, and impoverished pharmacokinetic profiles exhibited by conventional drugs have hindered the search for new entities. Plasmepsins, a group of *Plasmodium-*specific, aspartic acid protease enzymes, are involved in many key aspects of parasite biology, and this makes them interesting targets for antimalarial chemotherapy. Among different isoforms, PlmIX serves as an unexplored antimalarial drug target that plays a crucial role along with PlmV and X in the parasite’s survival by digesting hemoglobin in the host’s erythrocytes. In this study, fragment-based virtual screening was performed by modeling the three-dimensional structure of PlmIX and predicting its ligand-binding pocket by using the Sitemap tool. Screening identified the fragments with the XP docking score ≤ −3 kcal/mol from the OTAVA General Fragment Library (≈16,397 fragments), and the selected fragments were chosen for ligand breeding. The resulting ligands (≈69,858 ligands) were subsequently subjected to filtering based on the QikProp properties along with carcinogenicity testing performed using CarcinoPred-EL and then docked in the SP (≈14,078 ligands) as well as XP mode (≈3,104 ligands), and compared with that of control ligands 49C and I0L. The top-ranked ligands were taken further for the calculation of the free energy of binding using Prime MM–GBSA. Overall, a total of six complexes were taken further for MD simulation studies performed at 100 ns to attain a better understanding of the binding mechanisms, and compounds **3** and **4** were found to be the most efficient ones *in silico*. The analysis of compound **3** revealed that the carbonyl group present in position 1 on the isoindoline moiety (Arg554) was responsible for inhibitory activity against PlmIX. However, the analysis of compound **4** revealed that the amide linkage sandwiched between the phenyl ring and isoquinoline moiety (Lys555 and Ser226) as well as carbonyl oxygen of the carbamoyl group present at position 2 of the pyrazole ring (Gln222) were responsible for PlmIX inhibitory activity, owing to their crucial interactions with key amino acid residues.

## 1 Introduction

Malaria is a fatal parasitic infection that poses a significant risk to global health (Organization, 2022). Parasites belonging to the genus *Plasmodium* are the causative agents of the disease, which degrades human hemoglobin to obtain amino acids essential for their growth and maturation. Furthermore, the disease is transmitted through female Anopheles mosquitoes. Out of the four species, the most lethal human parasite is the *Plasmodium falciparum*, which contributes to the majority of deaths associated with malaria (Francis et al., 1997; Banerjee et al., 2002). The primary strategies to prevent and treat malaria comprise vector control and chemotherapy. However, there is an urgent concern about the widespread emergence of parasite drug resistance, especially to less expensive medications. *P. falciparum* has almost universally developed resistance to chloroquine drug, and there are certain sources of evidence of the declining effectiveness of widely available antimalarial medications such as sulfadoxine and pyrimethamine (Skinner-Adams et al., 2008). Therefore, it is urgently necessary to discover novel and unexplored anti-malarial drug targets to combat these variants. Plasmepsins (Plms), a subgroup of 10 aspartyl proteases identified in the *P. falciparum* genome, are the enzymes which initiate the breakdown of hemoglobin and have been extensively investigated as potential anti-malarial drug targets (Coombs et al., 2001). In the erythrocytic stages of *P. falciparum*’s life cycle, four Plms (PlmI, PlmII, PlmIV, and HAP) are expressed and localized in the food vacuole. These four Plms have received the majority of attention to date, but recent investigations have revealed that they are not necessary for parasite survival and further their inhibition alone would be insufficient (Florens et al., 2002; Bozdech et al., 2003; Le Roch et al., 2003). PMV, an endoplasmic reticulum integral membrane protein, may play a part in the parasite’s protein processing (Klemba and Goldberg, 2005). The remaining Plms (VI–X) have unknown functions, but Plms V, IX, and X are expressed during the blood stages of the malarial life cycle. Although they are expressed at the same time as PlmI to PlmIV, PlmV, PlmIX, and PlmX are not carried to the digestive vacuole (DV) and are therefore currently regarded as the primary targets of the discovered digestive vacuole plasmepsin inhibitors (Banerjee et al., 2002; Bozdech et al., 2003; Le Roch et al., 2003; Klemba and Goldberg, 2005). Among them, Plasmepsin IX or PlmIX could be considered an unexplored antimalarial drug target as it is an aspartyl protease which plays a crucial role in the parasite’s survival by digesting hemoglobin in the host’s erythrocytes, and inhibiting this enzyme could be a potential strategy to treat malaria and combat resistance (Nasamu et al., 2017). Owing to its crucial role, several studies have been performed to date on PlmIX inhibition. However, no candidate compound has been approved for clinical use because of the poor aqueous solubility, low bioavailability, and lack of target selectively (Pino et al., 2017; Cowman et al., 2021; Lisauskaitė et al., 2024).

In the present research initiative, we conducted homology modeling of PlmIX due to the absence of a resolved crystal structure for this protein. Additionally, fragment-based screening was applied to identify the key fragments binding within the active site of the modeled protein PlmIX to develop novel ligands, which were further taken for molecular docking, and the scores were compared with control ligands. Here, two control ligands were employed: control 49C (a peptidomimetic competitive inhibitor of PlmIX and PlmX) and control I0L (a potent dual inhibitor of PMIX and PMX that blocks the invasion of liver and blood stages and transmission to mosquitoes) (Pino et al., 2017; Cowman et al., 2021). Additionally, the obtained hits were also evaluated for their pharmacokinetic and toxicological parameters along with carcinogenicity testing. Binding free energies (BFE) were also calculated for the complexes of top-hit compounds with the modeled PlmIX protein using the MM–GBSA module. Finally, molecular dynamics simulation studies were also employed for the top compounds to evaluate the stability of the protein–ligand complex with an intention to develop PlmIX inhibitors that can potentially cure malaria and battle antimalarial resistance.

## 2 Materials and methods

### 2.1 Homology modeling of plasmepsin9

We performed homology modeling of PlmIX using the Prime application in Schrodinger Maestro Suite 2022-04 (Schrodinger, LLC, New York, NY, 2014). *P. falciparum* (isolate NF54) 627 amino acid sequence (accession ID: A0A2I0C265) was retrieved from the UniProt database. Using NCBI Protein BLAST against the PDB database, we identified Plasmepsin X protein of *Plasmodium vivax* 7TBD as the best template for the homology modeling of *P. falciparum* PlmIX, with a sequence similarity of 52.381% and gaps of 0.47619%. Thus, the three-dimensional crystal structure of Plasmepsin X protein of *P. vivax* (PDB ID: 7TBD) was retrieved from the RCSB PDB database for use as the template in the Schrodinger Prime modeling interface. The amino acids from GLU-199 to CYS-426 and LYS-487 to LEU-627 of *P. falciparum* PlmIX were only used for modeling as the corresponding template co-ordinates are available only for this region. Furthermore, the residues corresponding from MET-1 to ASN-198 and GLY-427 to LYS-487 were also not included for modeling as there is a break in the template structure as well in the region corresponding to these residues. Homology modeling was performed using the knowledge-based model building method in Prime, with one model being generated. The generated model was selected for further refinement, which comprised loop refinement and energy minimization using the OPLS4 force field in Schrodinger Prime (Roos et al., 2019). The minimized model was then validated using the Ramachandran plot, generated from PROCHECK at the SAVES version 4 server (Structure Analysis and Verification Server; http://services.mbi.ucla.edu/SAVES/). This model was optimized prior to docking using the protein preparation workflow in Schrodinger Maestro Suite 2022-04 (Schrodinger, LLC, New York, NY, 2014) (Jacobson et al., 2002; Jacobson et al., 2004; Madhavi Sastry et al., 2013).

### 2.2 Protein preparation and prediction of possible binding sites

Protein preparation was performed by the protein preparation module of Schrödinger suite 2022-04. Energy minimization of the protein structure was carried out using OPLS4 (Schrodinger, LLC, NY, United States, 2009) (Roos et al., 2019). Once the protein was ready, Sitemap was used to predict possible active sites (Halgren, 2007), addressing the inadequacy of experimental data regarding the active site of the protein from wet laboratory experiments. The predicted active sites were ranked based the on-site score and D-score. Sitemap identifies potential binding sites by linking together “site points” that are most likely to contribute to tight protein–ligand or protein–protein binding. These site points are useful for visualizing the extent of a site and can be employed to define the active site for virtual screening experiments using Glide (Halgren, 2007; Madhavi Sastry et al., 2013).

### 2.3 Fragment library preparation

In this study, the OTAVA general fragment library was used (retrieved from https://otavachemicals.com/products/fragment-libraries/general-fragment-library/). Fragment library was prepared by the LigPrep module of Schrodinger suite 2022-04 (Schrodinger, LLC, NY, United States, 2009). The 2D structures were transformed into 3D structures and optimized for geometry, followed by energy minimization and correction for chirality and desalting. The Epik module was used to generate ionization and tautomeric states between pH values of 5–9. The libraries were then subjected to minimization using the optimized potentials for liquid simulations-4 (OPLS-4) force field within the Schrodinger software. A single low energy confirmation per ligand was generated, and the optimized ligands were used for docking analysis (Madhavi Sastry et al., 2013).

### 2.4 Glide fragment docking

Receptor grid boxes were generated using the “Glide’s Receptor Grid Generation” module at the predicted active site of the protein. The prepared fragment library was docked against the binding pocket of the PlmIX protein using Glide and OPLS-4 force fields (Halgren et al., 2004; Roos et al., 2019). The algorithm identifies hydrogen-bonding, hydrophobic, and electrostatic interactions that are favorable while penalizing steric clashes. The minimized poses are then subjected to a re-scoring process using the GlideScore scoring function (Friesner et al., 2004). The docking process was done on two stages starting with standard precision (SP) docking and then with extra-precession (XP) docking (Friesner et al., 2006).

### 2.5 Fragment breeding

Fragments with the XP docking score ≤ −3 kcal/mol were selected for ligand breeding. The selected fragments were bred by using the BREED tool of the Schrodinger Maestro 2022-04 (Ho and Marshall, 1993). The created ligand libraries were prepared by the LigPrep module using the same parameter settings that have been used in the preparation of fragments’ libraries (Pierce et al., 2004; Madhavi Sastry et al., 2013).

### 2.6 *In silico* ADMET screening along with carcinogenicity testing

The ADMET properties of breed ligands were determined *in silico* using the QikProp module and then filtered using the ligfilter module of Schrödinger suite 2022-04. Eight filters were applied against the created ligands: the number of the violation to the Lipinski’s rule of five is 0, stars between 0 and 5, percent of human oral absorption is ≥80%, QPPCaco is ≥500, QPlog HERG is below −5, amine is in the range of 0–1, amidine is 0, and rtvFG is in the range of 0–2. The QikProp user manual was used to get the proper values for each property except for the rule of five where we opt for zero violation (Duffy and Jorgensen, 2000). The ligands were also tested for carcinogenicity by employing CarcinoPred-EL (Lipinski et al., 2012).

### 2.7 Glide ligand docking

The filtered ligands were docked against the predicted active site of the PlmIX protein using the Glide module (Friesner et al., 2004). The docking was carried out in two steps: standard precision (SP) docking and extra precision (XP) docking (Friesner et al., 2006). Large compounds of unknown quality are better screened by the SP docking mode. However, in contrast with SP docking, the XP docking mode offers more inclusive sampling and sophisticated scoring functions along with greater requirements for ligand–protein shape complementarity. Furthermore, the XP-Glide scores of the ligands were summarized and compared with the control ligands 49C and I0L.

### 2.8 Calculation of binding free energy using the Prime/MM–GBSA approach

The Prime module of Schrödinger suite 2022-04 was utilized to calculate the binding free energies of the ligand and receptor complex using the molecular mechanics–generalized born surface area (MM–GBSA) method. This involved the OPLS4 force field and VSGB solvent model, along with search algorithms (Li et al., 2011).

∆Gbind=∆GSA+Gsolv+∆EMM,

where ΔGSA denotes the difference between the surface area energies of the protein and the ligand along with the complexes, ΔGsolv represents the difference in the solvation energies of the complexes and the individual protein and ligands, and ΔEMM denotes the variation between the minimized energy of the protein–ligand complexes.

### 2.9 Molecular dynamics simulation

We employed the Desmond module of Schrödinger Release 2022-04 on the Linux system to evaluate the protein-binding interaction between our selected molecules and the Plasmepsin9 protein; the co-crystalized ligand of the template (I0L) and 49C (the best known inhibitor reported against PlmIX and PlmX) were taken as control, using molecular dynamic simulation. This was carried out to verify the structural integrity of the protein complex, using the optimized potentials for liquid simulation (OPLS4) force field at pH 7.4 (Roos et al., 2019). To identify better binding complexes, we performed 100 ns simulations, starting by solvating the chosen protein and selected complex with water molecules and providing boundaries to the complex with an orthorhombic box (Jorgensen and Madura, 1985; Lawrence and Skinner, 2003). We added Na+ and Cl− charges to neutralize charges and maintain a salt concentration of 0.15 M. The simulation was completed under a bar pressure of 1.01325 and a constant temperature of 300 K, with a mainlining recording interval of 5 ps (Lawrence and Skinner, 2003). We calculated the root mean square deviation (RMSD) of the protein based on the selected atom and evaluated the stability of the ligand–protein complex using RMSD and RMSF values. The ligand interactions with different atoms were determined for each trajectory frame. Additionally, we analyzed the radius of gyration to assess the structural compression during the 100 ns simulation.

## 3 Results and discussion

### 3.1 Molecular modeling of PlmIX

**FIGURE 1:**
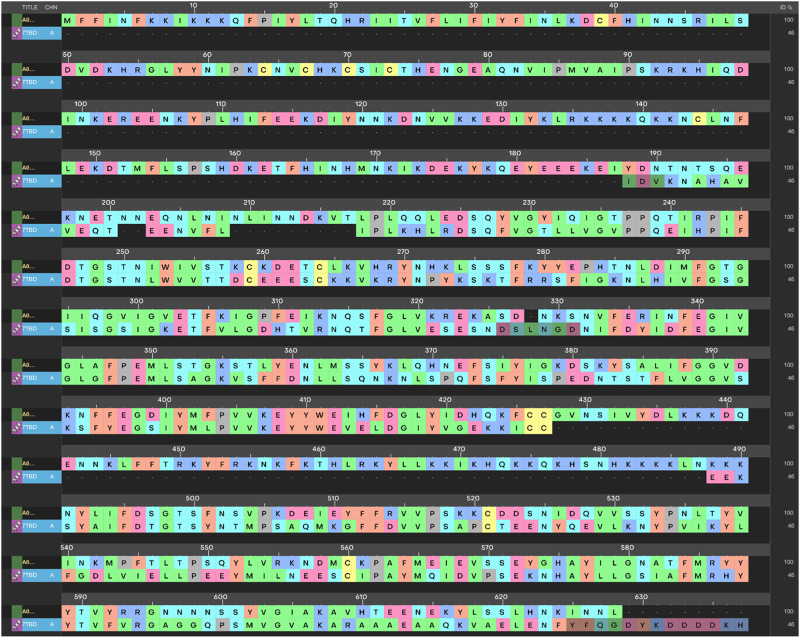
Sequence alignment of PlmIX FASTA sequence with PlmX of *P. vivax*.

Among the experimentally determined structures of the aspartyl proteases family, the PlmX protein of *P. vivax* 7TBD was found to be an appropriate template for PlmIX. The initial search for templates for the homology modeling of PlmIX through BLAST search revealed PlmX protein of *P. vivax* 7TBD as the best template for the homology modeling of *P. falciparum* PlmIX with a sequence identity of 52.381%. The sequence alignment between PlmIX of the *P. falciparum* and PlmX of the *P. vivax* is presented in Figure 1.

**FIGURE 2:**
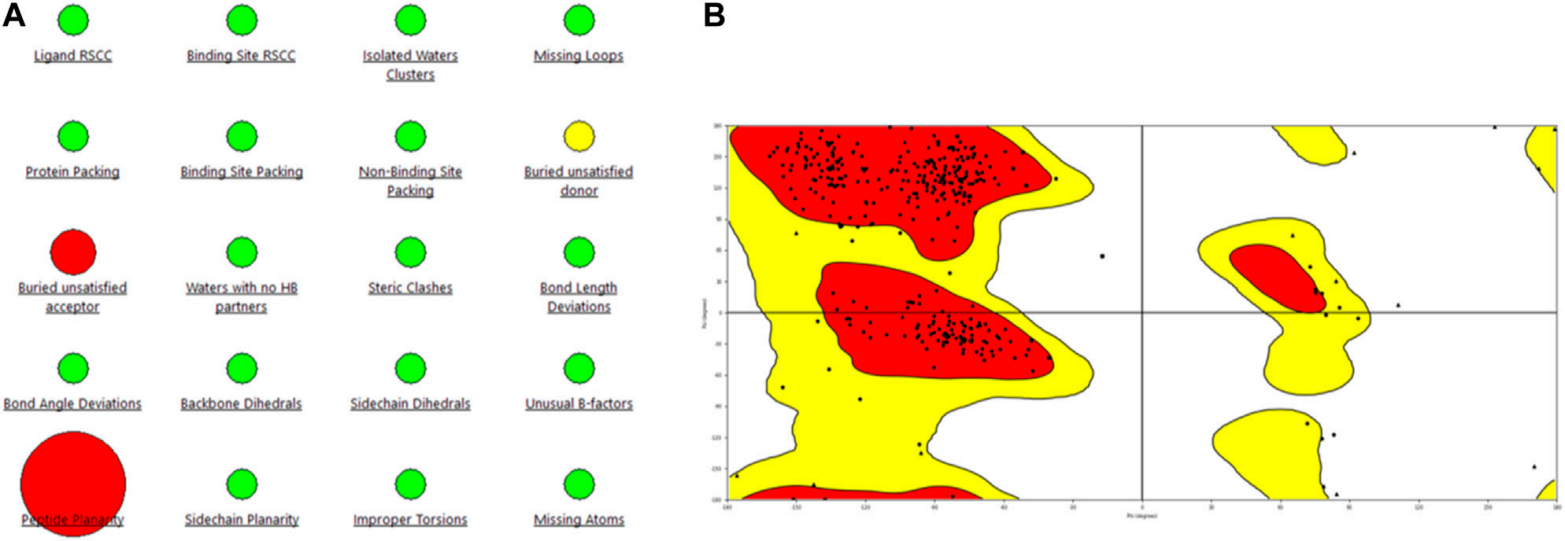
Representation of **(A)** protein reliability report and **(B)** Ramachandran plot of the model generated for the PlmIX protein.

Hence, the 3-dimensional structure of PlmX of *P. vivax* was used as the template for model generation. The model generated *via* the Schrodinger Prime was subjected to energy minimization and loop refinement processes by the Prime module of Schrodinger maestro. The generated model was validated *via* the Ramachandran plot displayed in Figure 2 along with the protein reliability report.

Statistical analysis of the plot revealed that 84.9% of the residues were in the most favored regions, with 13.3% in the additional allowed regions, 1.2% of the amino acids were in the generously allowed regions, and 0.6% of the residues were in the disallowed regions. These values support the structural validity of the modeled structure; hence, this structure was used for docking studies.

### 3.2 Analysis of molecular docking results

**FIGURE 3:**
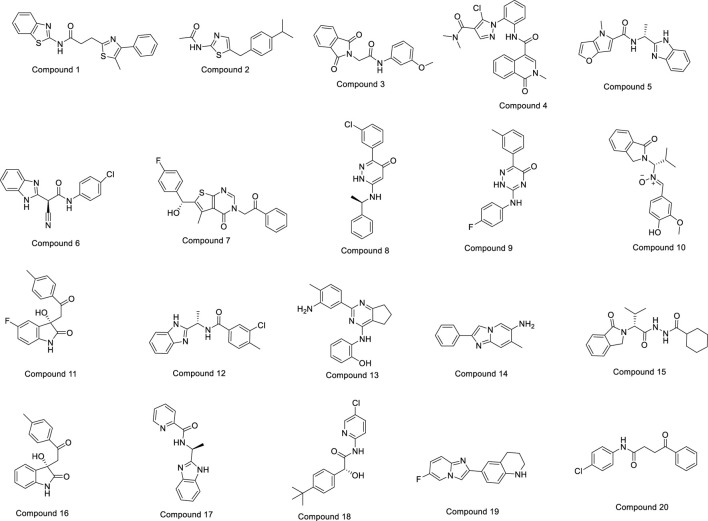
Chemical structures of the top 20 compounds.

The OTAVA fragment library was initially screened by standard precision SP molecular docking studies to find strong and potent inhibitors of the PlmIX protein and a therapeutic candidate to fight antimalarial resistance. Furthermore, the fragments with the XP docking score ≤ −3 kcal/mol were selected for ligand breeding, and subsequently, the resulting ligand library was employed for molecular docking. About 14,078 potential ligands were chosen subsequently for docking in the SP mode with the PlmIX protein. Out of them, only 3,104 ligands were chosen overall for docking in the XP mode based on the SP docking score and drug-likeness features. These compounds exhibited XP docking values ranging from −6.998 to 2.471 kcal/mol, which are detailed in Supplementary Material. Furthermore, based on the XP docking scores, the top **450** ligands were selected for binding free energy calculations using the Prime MM–GBSA approach. Among these ligands, only the top **20** ligands were selected for a thorough analysis of binding poses. The chemical structures of these top **20** compounds are displayed in Figure 3.

### 3.3 Evaluation of binding free energy calculation result

The Prime MM–GBSA method was employed for estimating the ligand BFE (ΔG_bind_) of 1–20/PlmIX-docked complexes. The measured BFE ranged between −101.62 and −70.49 kcal/mol (Table 1). It was found that the measured ΔG_bind_ was extremely suitable for the inhibitory effects of compounds at the binding site of the PlmIX protein and that it was closely connected with the glide energy component. The other key energy components included van der Waals energy (ΔG_vdW_: −72.52 to −26.17 kcal/mol), Coulombic energy (ΔG_cou_: −117.85 to 12.52 kcal/mol), hydrophobic energy (ΔG_lipo_: −31.69 to 17.64 kcal/mol), and covalent energy (−4.46 to 35.61 kcal/mol).

**TABLE 1:** Dossier evaluation of XP docking data for the top-scored ligands against PlmIX along with their BFE estimated utilizing the Prime MM–GBSA.

Compound	B_score_	G_score_	G_emodel_	G_energy_	G_evdW_	ΔG_bind_	ΔG_cov_	ΔG_lipo_	ΔG_vdW_	ΔG_cou_
Comp. **1**	7.11	−6.71	−52.60	−44.58	−40.68	−101.62	2.91	−19.95	−72.52	−13.36
Comp. **2**	10.36	−5.15	−48.46	−36.86	−31.10	−92.35	−2.67	−23.37	−40.59	−43.90
Comp. **3**	14.31	−5.71	−47.62	−37.64	−31.56	−88.08	20.64	−21.69	−42.63	−68.88
Comp. **4**	5.52	−5.59	−67.19	−47.73	−39.36	−87.13	42.80	−30.75	−48.38	−117.85
Comp. **5**	14.04	−5.62	−42.33	−36.76	−31.10	−85.91	16.80	−17.64	−67.41	−42.63
Comp. **6**	5.73	−5.49	−54.55	−39.76	−28.48	−84.9	20.98	−19.25	−34.8	−73.63
Comp. **7**	10.44	−6.36	−53.03	−42.81	−36.46	−83.82	23.52	−26.17	−47.95	−79.68
Comp. **8**	13.91	−5.33	−53.57	−40.83	−27.48	−78.3	38.64	−31.69	−53.12	−61.54
Comp. **9**	6.40	−5.25	−48.66	−39.63	−25.81	−77.81	15.59	−19.71	−37.78	−67.07
Comp. **10**	15.04	−6.99	−51.29	−43.05	−29.90	−77.65	15.93	−26.38	−42.61	−24.79
Comp. **11**	5.69	−5.17	−43.31	−34.02	−27.29	−77.6	20.47	−25.33	−37.5	−52.87
Comp. **12**	5.31	−5.57	−43.75	−35.41	−30.49	−75.85	20.04	−20.98	−55.01	−55.33
Comp. **13**	7.61	−6.34	−52.71	−37.16	−25.53	−75.51	35.61	−18.49	−26.52	−99.39
Comp. **14**	5.20	−5.19	−35.27	−26.72	−19.37	−75.12	29.50	−22.35	−65.37	−79.65
Comp. **15**	11.00	−5.63	−49.10	−37.81	−30.11	−74.49	−4.46	−24.45	−49.2	−47.44
Comp. **16**	5.63	−5.21	−40.93	−33.10	−26.15	−73.41	29.62	−20.84	−27.92	−65.47
Comp. **17**	13.18	−5.52	−41.71	−33.53	−28.89	−72.98	10.38	−18.62	−51.38	22.08
Comp. **18**	15.91	−5.21	−42.07	−33.99	−29.33	−72.73	−3.83	−22.08	−48.80	12.52
Comp. **19**	4.78	−5.26	−36.93	−29.10	−26.28	−72.68	33.79	−20.34	−57.49	−72.86
Comp. **20**	10.41	−5.16	−44.32	−34.27	−29.23	−70.49	−1.01	−21.29	−33.13	−25.34
Control **49C**	—	−4.32	−60.70	−45.23	−37.25	−65.82	23.5	−26.72	−58.12	−34.00
Control **I0L**	—	−4.03	−54.43	−41.99	−35.27	−48.66	17.69	−20.46	−43.12	−17.59

B_score_, breed score; G_score_, GlideScore (kcal/mol); G_emodel_, Glide model (kcal/mol); G_energy_, Glide energy (kcal/mol); G_evd_, Glide van der Waals energy (kcal/mol); ΔG_bind_, free energy of binding (kcal/mol); ΔG_cov_, covalent energy (kcal/mol); ΔG_lipo_, hydrophobic energy (kcal/mol); ΔG_vdW_, van der Waals energy; ΔG_cou_, Coulombic energy (kcal/mol).

**FIGURE 4:**
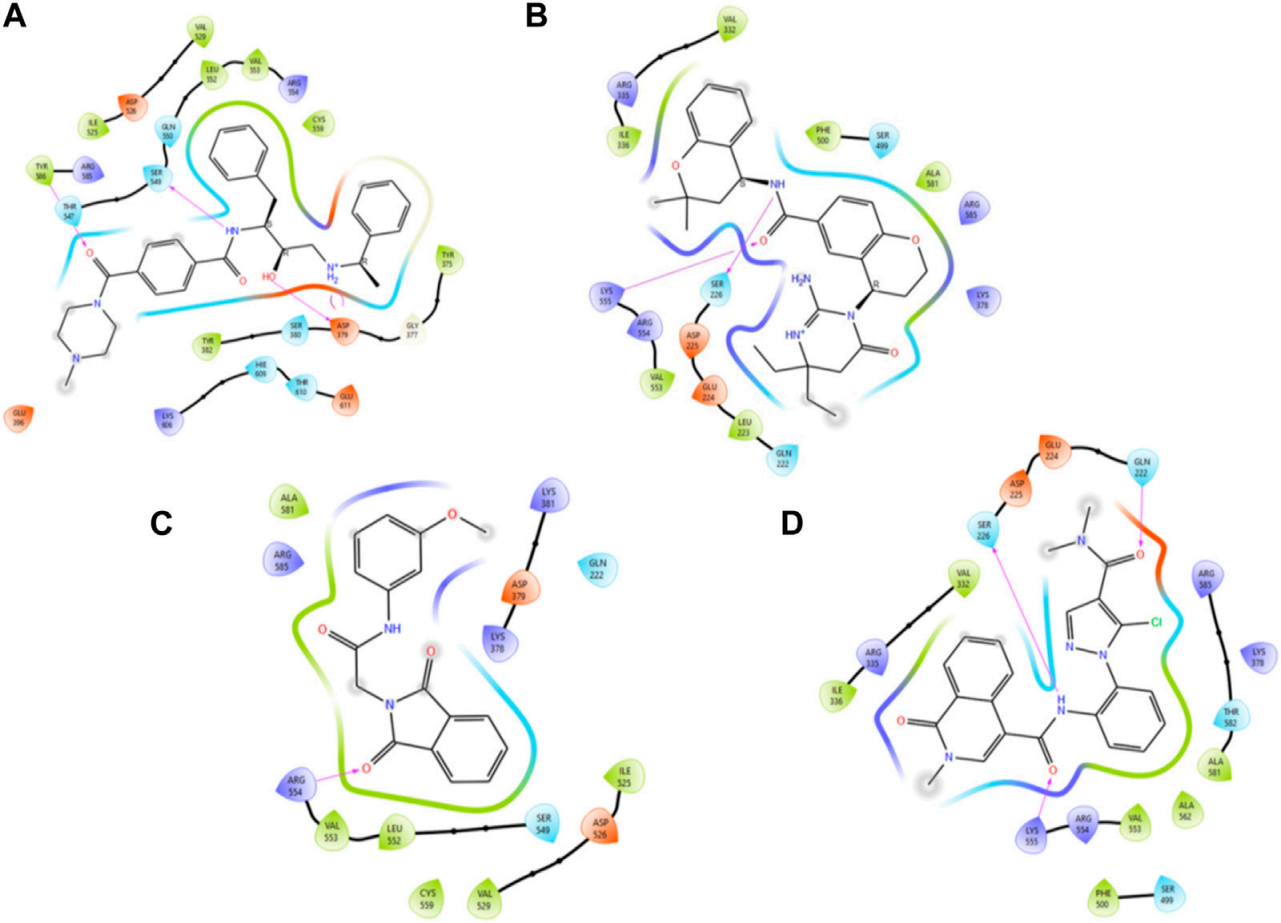
Illustration of the two-dimensional protein–ligand contacts exhibited by **(A)** control ligand 49C, **(B)** control ligand I0L, **(C)** compound **3**, and **(D)** compound **4** based on MM–GBSA evaluation.

In accordance with the outcomes and findings of XP docking and BFE estimations, it was revealed that the H-bonds, π-cationic bonds, and salt bridges formed with the amino acid residues including Gln222, Ser226, Lys378, Asp379, Ser549, Arg554, Lys555, and Arg585 within the projected docking site of PlmIX, which was predicted by the Sitemap tool of Schrödinger. Among the top 20 compounds subjected to a thorough analysis of binding poses, it was revealed that compounds 3 and 4 possessed better binding interactions with the amino acid residues within the active pocket of the PlmIX protein along with good docking scores and BFE values. The 2D protein–ligand contacts for both the controls (49C and I0L) and compounds 3 and 4 on the basis on MM–GBSA evaluation are illustrated in Figure 4.

**FIGURE 5:**
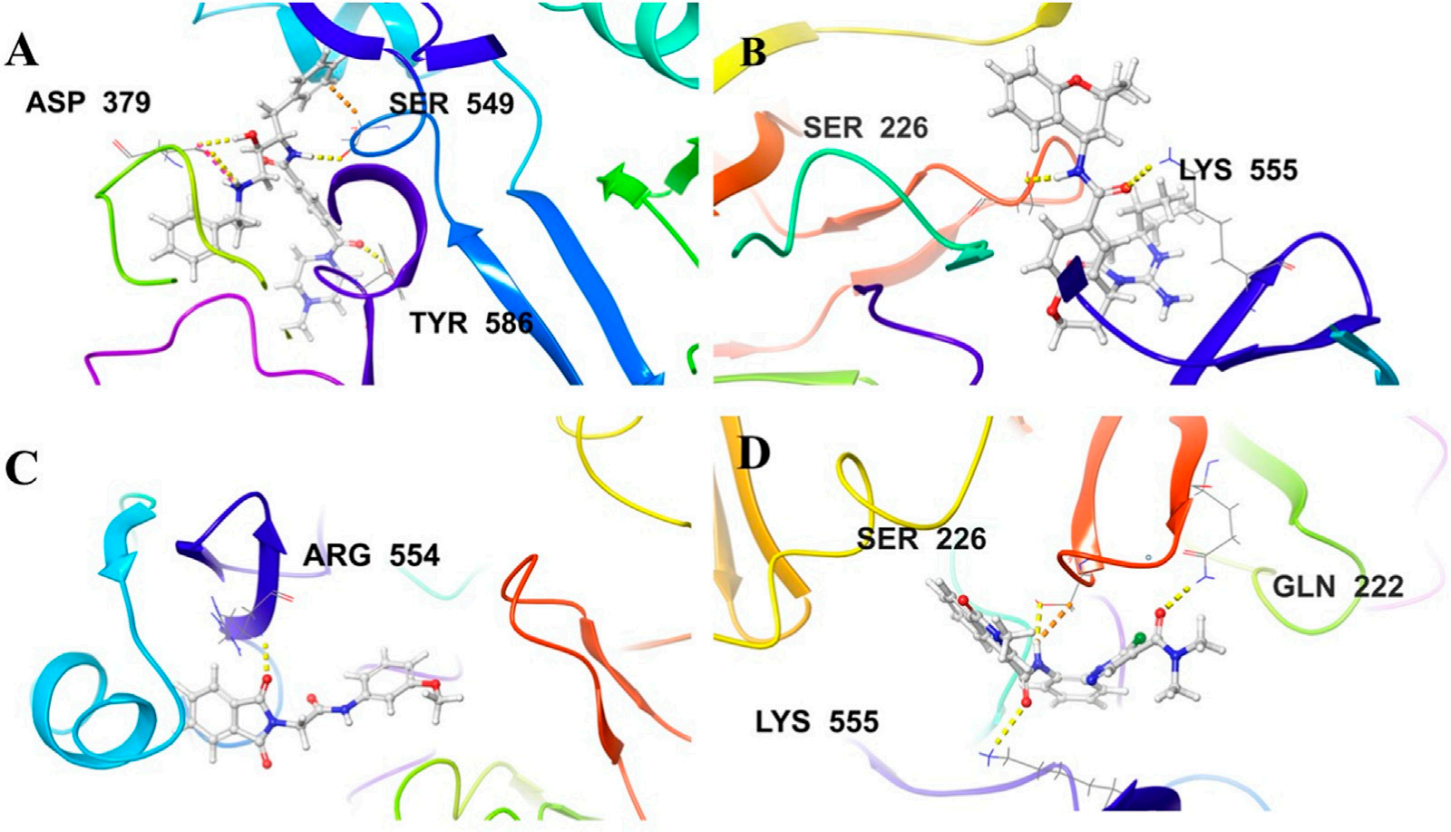
Illustration of the 3D-contacts formed by **(A)** Control ligand 49C **(B)** Control ligand I0L **(C)** Compound 3 and **(D)** Compound 4 with the key amino acid residues present in the predicted active site of PlmIX protein.

Control ligand **49C** (Figure 5A) possessed a G_score_ value of −4.32 kcal/mol against the PlmIX protein and subsequently was also subjected to post-docking interaction analysis. Overall, this control ligand formed three hydrogen bonds with Tyr586, Ser549, and Asp379 amino acid residues of the PlmIX protein. The carbonyl oxygen present at position 1 of the piperazine ring of the control ligand accepted one H-bond from the–NH_2_ group of Tyr586 (∼C=O⋯H–N–H, 1.746 Å). The –NH group further attached with the same carbonyl group of the piperazine ring donated one H-bond to the carbonyl group of Ser549 amino acid residue (∼NH⋯O=C–OH, 1.73 Å). Another H-bond was seen between the –OH group and the Asp379 amino acid residue (∼OH⋯O=C–OH, 2.010 Å). Two more H-bonds could be seen between the ∼^+^NH_2_ group and the same Asp379 (∼NH⋯O=C–OH, 2.302 Å) and (∼NH⋯O=C–OH, 2.310 Å).

The second control ligand (**I0L**) (Figure 5B) also displayed an almost similar G_score_ value (−4.03 kcal/mol), and its binding interactions with the PlmIX protein were also assessed and compared with control ligand 49C. It formed only two H-bonds with Ser226 and Lys555 amino acid residues. The –NH group attached at the 1st position of the chromanyl ring of the control ligand donated one H-bond to the carbonyl functional group of the Ser226 amino acid (∼NH⋯O=C–OH), 1.840 Å). The 2^nd^ H-bond could be seen between the carbonyl group attached with the same –NH group of the chromanyl ring and the –NH group of Lys555 (∼C=O⋯H–N–H), 2.235 Å).

In accordance with the docking and BFE analysis, compound **3** obtained a remarkably more favorable and greater G_score_ value (5.71 kcal/mol) against the PlmIX protein in contrast with both the control ligands and also displayed one hydrogen bonding interaction (Figure 5C). The carbonyl oxygen present at position 1 of the isoindoline moiety accepted one H-bond from the –NH_2_ group of the Arg554 amino acid residue (∼C=O⋯H–N–H, 1.72 Å).

In comparison with both the control ligands, compound **4** (Figure 5C) possessed a remarkably greater G_score_ value (−5.59 kcal/mol) and also formed similar H-bonds with the key amino acid residues including Gln222, Ser226, and Lys555 (Figure 5). The carbonyl oxygen of the dimethyl carbamoyl group attached to the pyrrole ring displayed one H-bond with Gln222 (∼C=O⋯H–N–H), 1.769 Å). Moreover, one more H-bond could be seen between another carbonyl oxygen of the amide group attached to the isoquinoline ring of compound 4 and the –NH group of Lys555 (∼C=O⋯H–N–H), 2.256 Å). The –NH group of the same amide moiety formed another H-bond with the carbonyl oxygen of the Ser226 (∼NH⋯O=C–OH), 1.821 Å).

### 3.4 Prediction of ADMET parameters and *in silico* carcinogenicity

vUtilizing the QikProp approach, the absorption, distribution, metabolism, excretion, and toxicity (ADMET) parameters were assessed to determine the appropriateness of the selected ligands. The outcome of the ADMET evaluation (Table 2) demonstrated that chosen compounds **1–20** possess remarkable pharmacological drug potential and also follows the Lipinski’s rule of five with 0 violations. The donating H-bonds were found between 0 and 3.5 (ideal value: 0–6), whereas the acceptor H-bonds were found between 1 and 9.5 (ideal value: 2–20). These ligands further displayed remarkable oral absorption (QPlogP_o/w_ varying from −2.518 to 4.31) and Caco-2 cell permeability (QPPCaco) ranging from −136.161 to 443.194), which are in compliance with the suggested ranges (−2–6.5) and (>500), respectively. As evidenced by QPlogBB values varying from −0.06 to −0.92 (ideal range: −3.0 to 1.2), these ligands also illustrated optimal blood–brain barrier (BBB) permeability. In addition, the optimal CNS activity of the following compounds was also revealed by the values that varied between 0 and −1 (ideal values: −2 to +2). Moreover, these top compounds were also screened and tested for *in silico* carcinogenicity parameters employing CarcinoPred-EL. Among these compounds, only compound **14** was found to be carcinogenic in nature.

**TABLE 2:** Dossier on insights into the ADMET parameters of the ligands **1-20**.

Compound	SASA	Donor HB	Accpt HB	QPlog P_o/w_	QPlog HERG	QPP Caco	QPlogBB	QPlogK_p_	RF	CNS	*In silico* carcinogenicity testing
Comp. **1**	685.49	1	5.5	4.70	−6.81	2,170.96	−0.20	−1.03	0	0	Non-carcinogen
Comp. **2**	580.39	1	4	3.47	−5.24	1,784.50	−0.36	−2.01	0	0	Non-carcinogen
Comp. **3**	581.92	1	6.25	2.37	−5.93	623.31	−0.92	−2.32	0	−1	Non-carcinogen
Comp. **4**	698.29	1	9.5	3.10	−6.01	517.69	−0.81	−2.52	0	−1	Non-carcinogen
Comp. **5**	573.74	2	4.5	3.43	−5.81	2,661.57	−0.19	−1.13	0	0	Non-carcinogen
Comp. **6**	566.21	1	4.5	3.19	−5.92	592.82	−0.74	−2.29	0	−1	Non-carcinogen
Comp. **7**	688.04	1	7.7	3.56	−6.58	644.99	−0.90	−1.9	0	−1	Non-carcinogen
Comp. **8**	607.96	2	5.5	3.15	−6.12	843.67	−0.63	−2.04	0	0	Non-carcinogen
Comp. **9**	569.70	2	5.5	2.49	−5.90	735.81	−0.67	−2.31	0	0	Non-carcinogen
Comp. **10**	651.37	1	5.5	3.81	−5.83	1,013.97	−0.90	−1.78	0	−1	Non-carcinogen
Comp. **11**	546.73	1	4.25	3.01	−5.26	512.81	−0.82	−2.72	0	−1	Non-carcinogen
Comp. **12**	590.12	2	4	3.94	−5.71	2,624.57	−0.06	−1.32	0	0	Non-carcinogen
Comp. **13**	628.57	3.5	4.25	3.43	−5.83	857.52	−0.85	−2.11	0	−1	Non-carcinogen
Comp. **14**	476.78	1.5	2.5	2.93	−5.29	2,189.72	−0.11	−1.55	0	0	Carcinogen
Comp. **15**	672.94	0.5	6.5	3.66	−5.33	1,043.87	−0.77	−2.32	0	−1	Non-carcinogen
Comp. **16**	537.33	1	4.25	2.67	−5.36	514.72	−0.92	−2.58	0	−1	Non-carcinogen
Comp. **17**	544.79	2	5	2.54	−5.98	1,301.76	−0.51	−1.65	0	0	Non-carcinogen
Comp. **18**	602.34	2	5.2	3.39	−5.39	1,184.02	−0.50	−2.04	0	0	Non-carcinogen
Comp. **19**	523.48	1	2.5	3.95	−5.38	4,207	0.31	−1.23	0	1	Non-carcinogen
Comp. **20**	574.82	1	4.5	3.37	−6.21	1,462.82	−0.44	−1.40	0	0	Non-carcinogen

SASA, total solvent accessible surface area (Å^2^); Donor HB, estimated number of hydrogen bonds donated by the solute to water molecules; Accpt HB, estimated number of hydrogen bonds accepted by the solute from water molecules; QPlogP_o/w_, predicted octanol/water partition coefficient; QPlog HERG, predicted IC_50_ value for the blockage of HERG K^+^ channels; QPPCaco, predicted apparent Caco-2 cell permeability; QPlogBB, predicted brain/blood partition coefficient; QPlogKp, predicted skin permeability; RF, number of violations of Lipinski’s rule of five; CNS, predicted central nervous system activity.

### 3.5 Assessment and evaluation of molecular dynamic simulation studies

**FIGURE 6:**
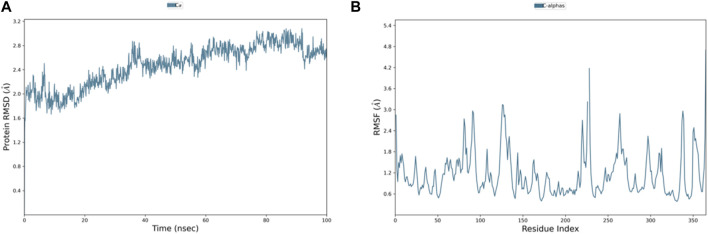
Representation of **(A)** RMSD and **(B)** RMSF graphs of the PlmIX Apoprotein throughout 100 ns MD simulation analysis.

Among the top-ranked 20 compounds, six complexes were opted for the MD simulation analysis as a result of their better docking score, remarkably more appealing binding free energy, and favorable results of ADMET parameters and carcinogenicity testing. MD simulations were performed for the six complexes, and among them, the complexes of compounds 3 and 4 with PlmIX were found to exhibit better results; hence, it was subsequently contrasted with the Apoprotein, control 49C/PlmIX, and control I0L/PlmIX complexes with the objective to attain a better understanding of the binding mechanisms. Evaluating the 100 ns MD trajectory frames facilitated the analysis of the reliability and constancy of the simulated positions. The RMSD of PlmIX Apoprotein progressively rose from the beginning of the simulation time, and it showed values within a range of 1.7–2.4 Å at the first 40 ns of the simulation time. After that, a slight increment has been observed in the RMSD value to be in the range of 2.4–3 Å till the end of the simulation time (Figure 6A).

**FIGURE 7:**
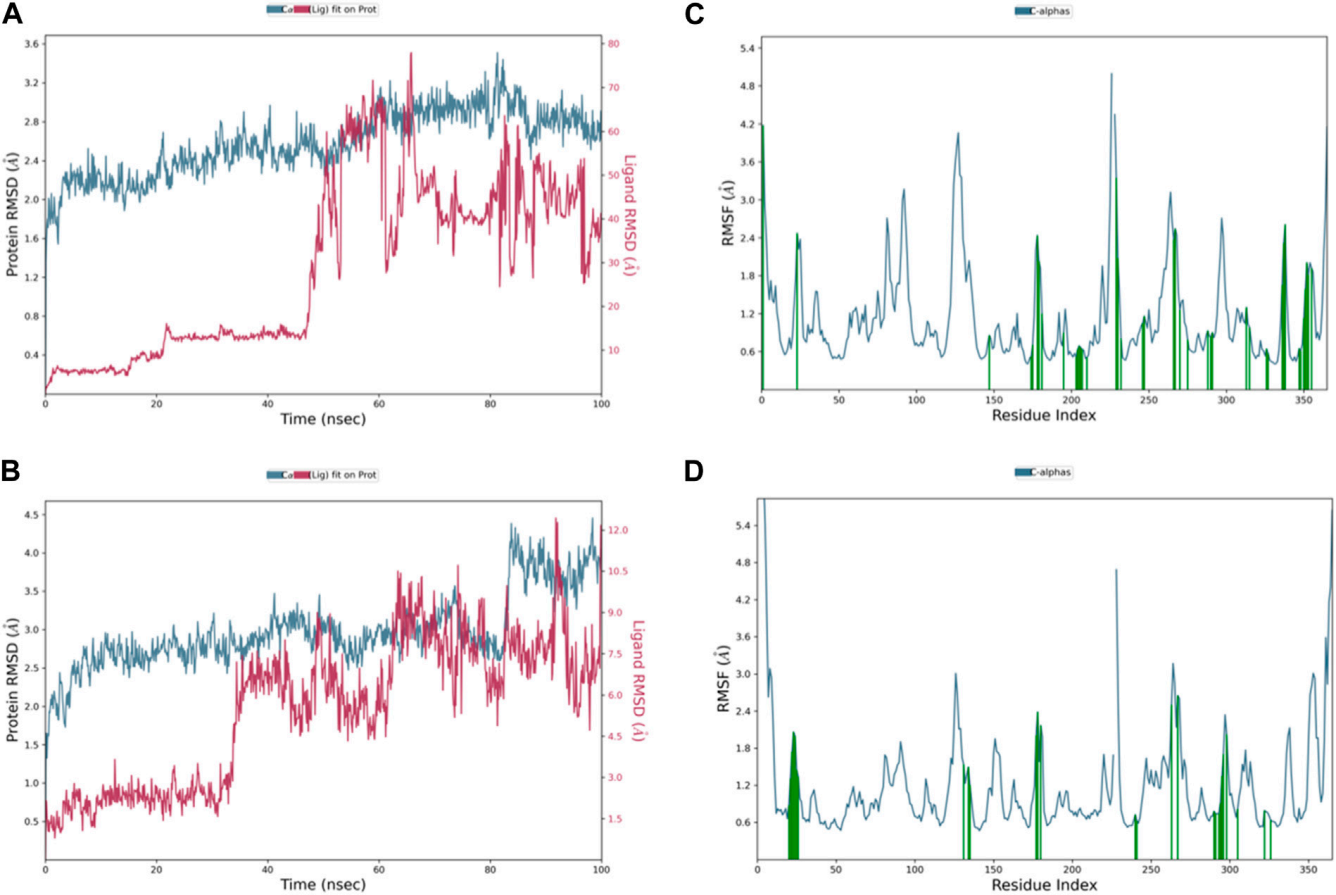
Representation of **(A)** control 49C’s RMSD graph, **(B)** control I0L’s RMSD graph, **(C)** control 49C’s RMSF graph, and **(D)** control I0L’s RMSF graph throughout 100 ns MD simulation.

The RMSD plot for the control ligand 49C is displayed in Figure 7A, demonstrating relatively similar values with the Apoprotein which were in the range of 2.5 Å up to 50 ns of the simulation time except for a few sharp spikes reached up to 2.8 and 3.0 Å at 32 and 40 ns, and RMSD then slightly increased to be in the range of 2.8–3 Å till the end of the simulation. The only significant difference observed was the high RMSD fluctuation in the 80–85 ns where it reached up to 3.6 Å and decreased to return to the previous range. In addition, we can observe that the RMSD values of 49C were relatively low and fell within the accepted range for the first 10 ns followed by one fold increment and stabilization up to 50 ns of the simulation time, but there was a sudden sharp increment in the ligand RMSD values which suggests that the ligand came out of the binding pocket of the protein.

Although the complex of control I0L with PlmIX originally had a remarkably stable RMSD value ranging from 2.5 to 3 Å (Figure 7B), it was observed that after 80 ns, the RMSD graph abruptly ascended to 4.5 Å and stayed constant till the end of the simulation. Furthermore, it has been observed that the RMSD values of I0L were relatively lower than those of 49C; they were in the range of 1.8–2.8 Å for the first 30 ns, but there was a sudden sharp increment in the ligand RMSD values accompanied by many strong fluctuations which suggests that the ligand came out of the binding pocket of the protein.

**FIGURE 8:**
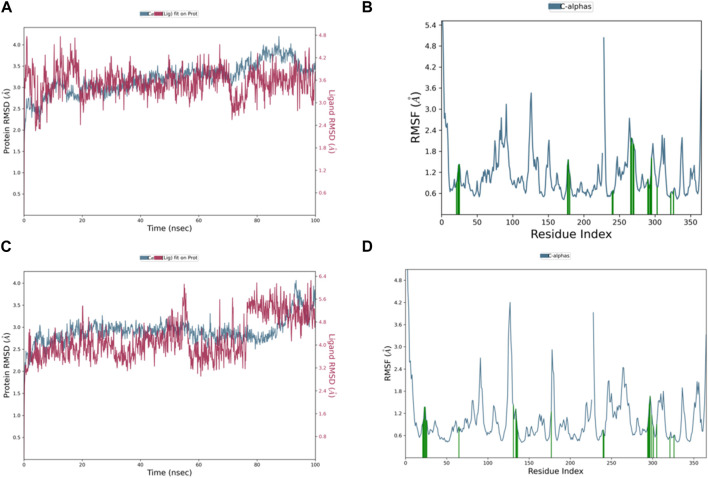
Representation of **(A)** RMSD graph of compound **3**, **(B)** RMSF graph of compound **3**, **(C)** RMSD graph of compound 4, and **(D)** RMSF graph of compound 4 during 100 ns MD simulation.

The RMSD graph in Figure 8A illustrates the evolving behavior of compound 3 as it moves from its initial position to its final location during the simulation. Initially, the RMSD steadily approached equilibrium, stabilizing at approximately 2.5–3.2 Å. However, around 80 ns, there was a slight increase in the RMSD, reaching 4.0 Å and lasting for 9 ns. Following this increase, the RMSD decreased and returned to the previously mentioned equilibrium values, maintaining this stability until the end of the trajectory. The range of RMSD values for the complex suggests a stronger binding than both control groups. Notably, compound 3 exhibited lower ligand RMSD values than both control ligands, indicating a more stable interaction with minimal fluctuations, indicating that the ligand remained within the protein binding pocket throughout the entire simulation.

The dynamic behavior of compound 4 from the original spot to the ultimate spot during the course of the simulation depicted by the RMSD graph is presented in Figure 8C. The RMSD sailed in the direction of the equilibrium from the onset, reaching it at 2.8 to 3.0 Å and holding the same level until there was a sudden increment around 90 ns up to 4.0 Å; this increment lasted for 6 ns only and was followed by a decline reaching the same equilibrated system values as mentioned earlier till the end of the trajectory. The range of RMSD values of the complex indicates stronger binding than both controls. Interestingly, compound 4 exhibited RMSD values lower than both control ligands, and the system was more stable with minimum fluctuations, which means that the ligand remained in the protein-binding pocket for the entire time of the simulation.

Additionally, the flexibility of the protein’s framework is also crucial for several biological activities, such as ligand recognition, protein interactions, bonding rigidity, and stiffness. Therefore, we investigated the flexibility of amino acid residues using RMSF graphs. The RMSF plot for the PlmIX Apoprotein is represented in Figure 6B. The control 49C’s RMSF (Figure 7C) revealed that many of the residues contributing to the binding with the ligand have high RMSF values such as GLU-224, LYS-378, ASP-379, SER-380, LYS-487, LYS-488, and ILE-525, which showed the RMSF values of 2.47, 2.21, 2.44, 2.03, 4.35, 3.35, and 2.37 Å, respectively. The RMSF graph of control I0L (Figure 7D) showed high RMSF values for the residues that bind to the ligands during the simulation time. For instance, GLU-224, ASP-225, LYS-378, ASP-379, LYS-381, ASP-522, ASP-526, ASN-556, and ASP-557 showed RMSF values of 2.06, 1.99, 2.01, 2.38, 2.17, 2.50, 2.65, 2.34, and 2.03, respectively. These results indicate that the PlmIX binding pocket is highly unstable.

The RMSF chart for compound 3 (Figure 8B) reveals that the majority of the contributing residues within the system exhibit relatively modest RMSF values, falling in the range of 0.48–1.56 Å. Notably, the key residues, LEU-552 and ARG-585, which engage in hydrogen bonding with the ligands, demonstrate low RMSF values of 0.86 and 0.68 Å, respectively. It is worth mentioning that ARG-585 also forms a stable Pi–cation interaction that persists for approximately 75% of the simulation duration. Additionally, VAL-553 and ALA-562, which engage in relatively strong hydrophobic interactions, exhibit RMSF values of 1.09 and 0.78 Å, respectively. These findings indicate that compound 3 fits exceptionally well within the binding pocket of PlmIX.

The RMSF plot of the compound 4 (Figure 8D) complex displayed that none of the highly fluctuating residues are contributing to the system, and the highest RMSF values were exhibited by LYS-555, ASN-556, GLU-224, and ASP-225, which showed values of 1.46, 1.67, 1.37, and 1.37 Å, respectively. Furthermore, the most important residues in the system are GLN-222 and SER-226, which formed H-bonds with the ligand, showing RMSF values of 0.92 and 0.99 Å, respectively. In addition, ARG-335, PHE-500, and LYS-555 which formed hydrophobic interactions with the ligand exhibited RMSF values of 1.32, 0.64, and 1.46 Å, respectively, thus indicating that PLMIX is more stable when binding to compound 4 than control ligands.

**FIGURE 9:**
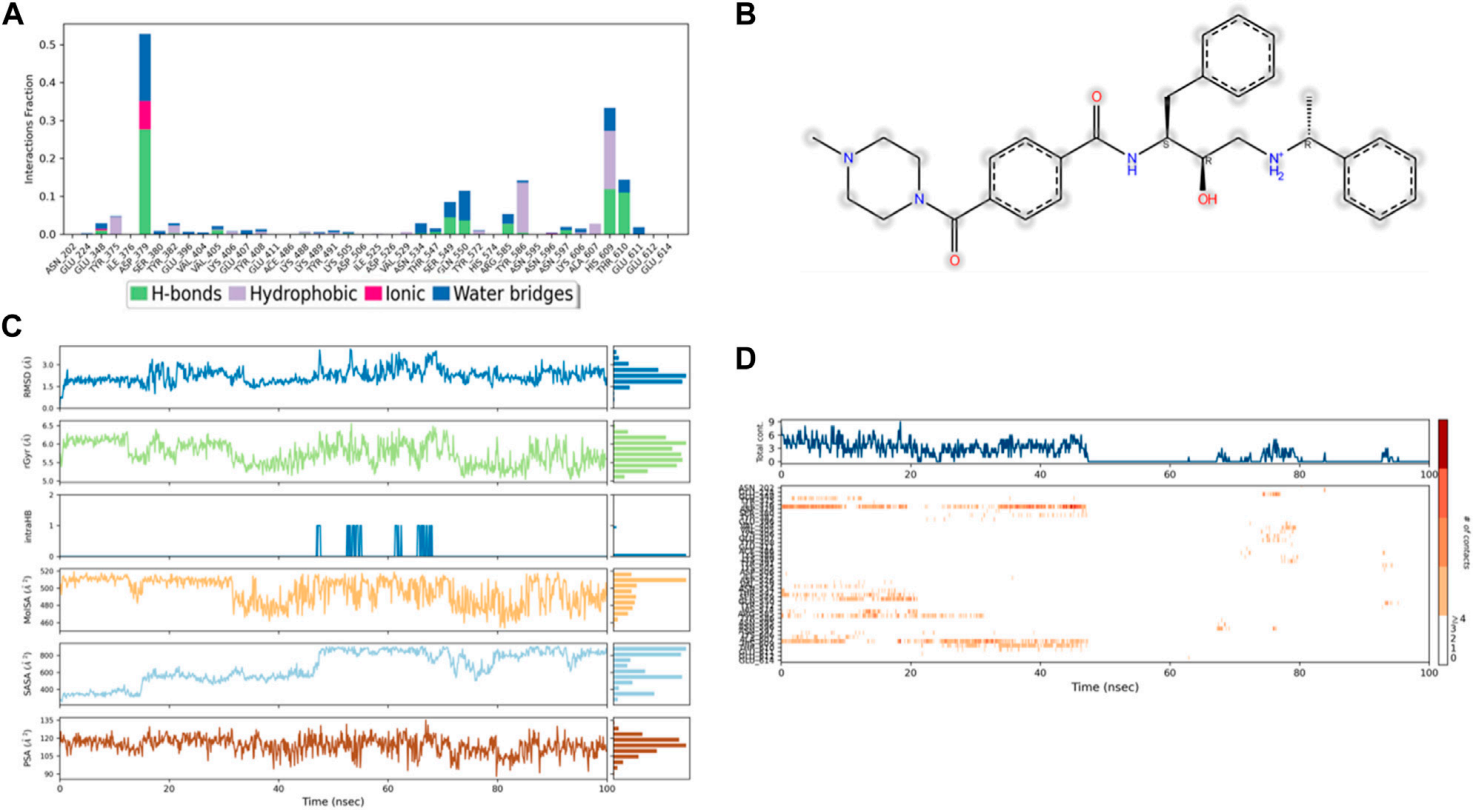
**(A)** Protein interaction with control 49C, **(B)** a schematic of detailed ligand–atom interactions with the protein residues, **(C)** ligand properties such as ligand RMSD, radius of gyration, intermolecular H-bonds, molecular surface area, solvent accessible surface area, and polar surface area, and **(D)** a timeline representation of the interactions and contacts (H-bonds, hydrophobic, ionic, and water bridges).

**FIGURE 10:**
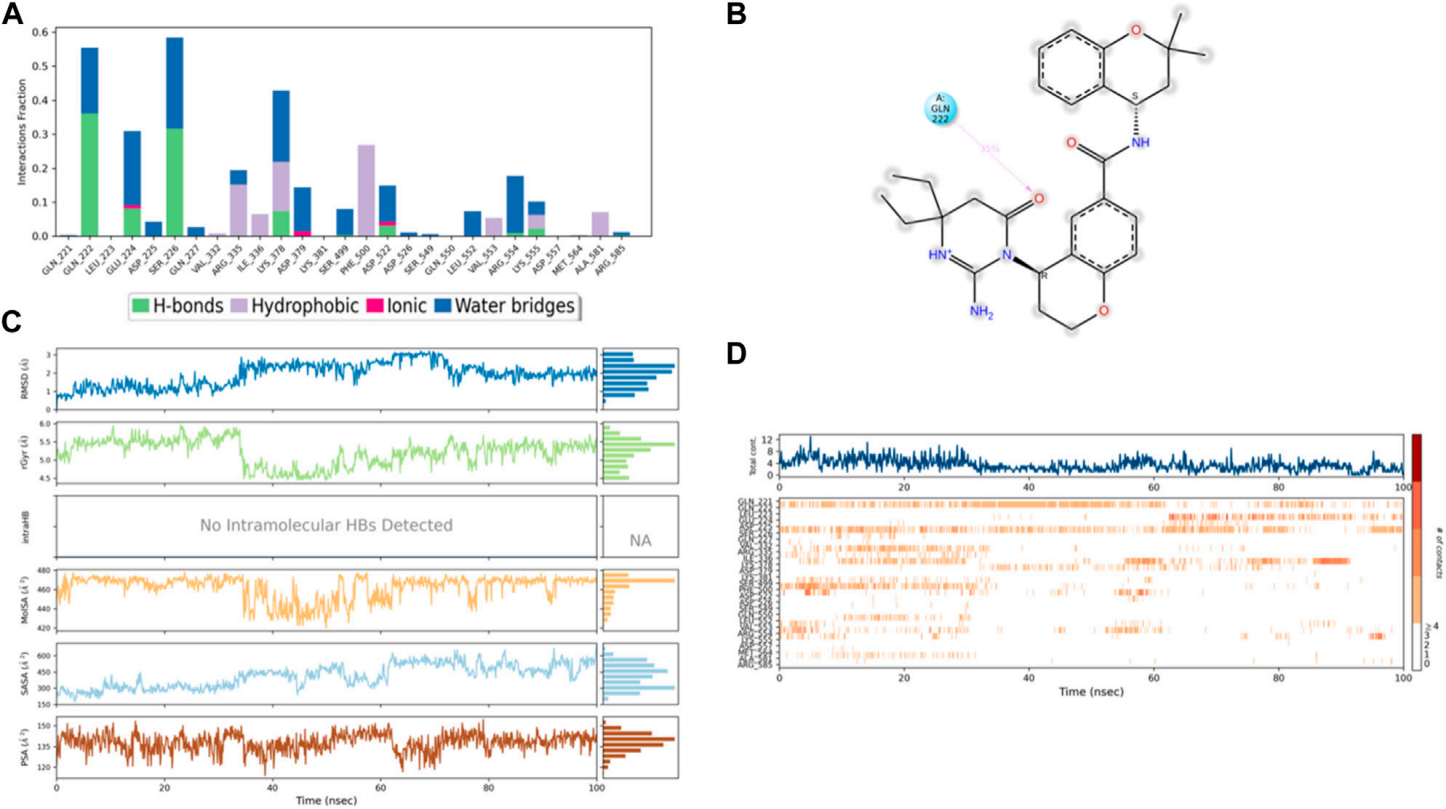
**(A)** Protein interaction with control I0L, **(B)** a schematic of detailed ligand–atom interactions with the protein residues, **(C)** ligand properties such as ligand RMSD, radius of gyration, intermolecular H-bonds, molecular surface area, solvent accessible surface area, and polar surface area, and **(D)** a timeline representation of the interactions and contacts (H-bonds, hydrophobic, ionic, and water bridges).

**FIGURE 11:**
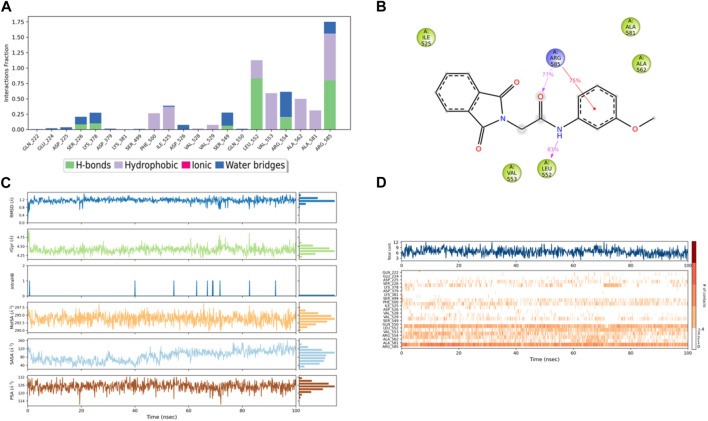
**(A)** Protein interaction with compound **3**, **(B)** a schematic of detailed ligand–atom interactions with the protein residues, **(C)** ligand properties such as ligand RMSD, radius of gyration, intermolecular H-bonds, molecular surface area, solvent accessible surface area, and polar surface area, and **(D)** a timeline representation of the interactions and contacts (H-bonds, hydrophobic, ionic, and water bridges).

**FIGURE 12:**
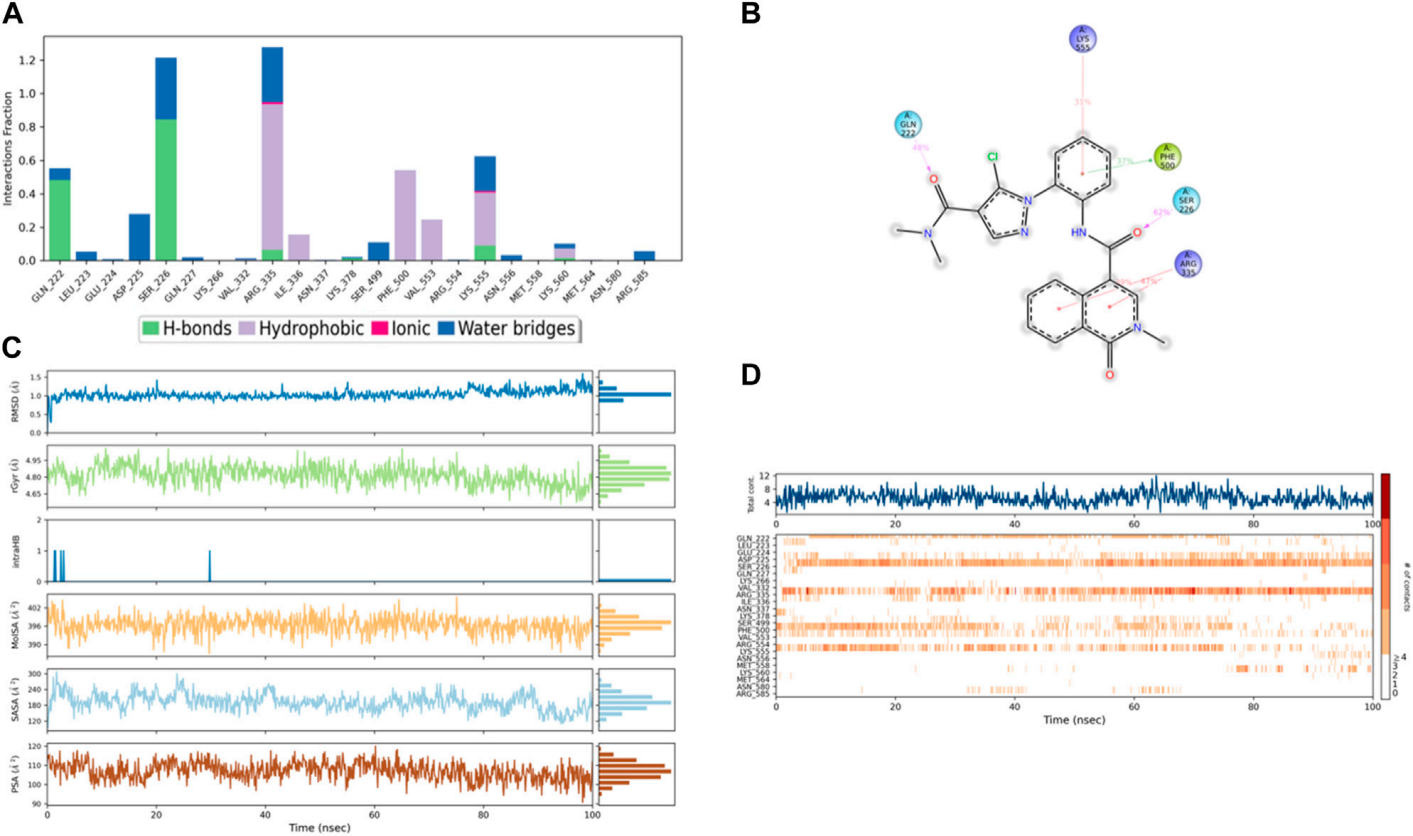
**(A)** Protein interaction with compound **4**, **(B)** a schematic of detailed ligand–atom interactions with the protein residues, **(C)** ligand properties such as ligand RMSD, radius of gyration, intermolecular H-bonds, molecular surface area, solvent accessible surface area, and polar surface area, and **(D)** a timeline representation of the interactions and contacts (H-bonds, hydrophobic, ionic, and water bridges).

The ligands’ compactness was evaluated *via* the radius of gyration, and rGyr values of the controls 49°C (Figure 9C) and I0L (Figure 10C) were in the range of 5.2–6.2 Å and 5.2–5.7 Å in most of the trajectory time. In contrast, compound 3 (Figure 11C) and compound 4 (Figure 12C) exhibited lower rGyr values throughout the simulation between the range of 4.25–4.50 and 4.6 to 4.9 Å, respectively; this indicates that compound 3 and compound 4 complexes are well compacted as compared to both controls.

Solvent accessible surface area (SASA) analysis shows that 49C control (Figure 9C) has relatively high SASA values that ranged from 340 to 400 Å^2^ in the first 15 ns of the trajectory time, and then SASA values elevated to be in the range of 500–600 Å^2^ till 45 ns of the trajectory time. Eventually, the ligand showed SASA values in the range of 800–900 Å^2^ till the end of the simulation. In addition, control I0L showed relatively lower SASA values (Figure 10C), which ranged from 280 to 380 Å^2^, in the first 45 ns of the trajectory time and then the values increased to be in the range of 450–600 Å^2^. On the contrary, compounds 3 and 4 showed significantly lower SASA values that mostly ranged from 60 to 120 Å^2^ and 150 to 220 Å^2^, respectively, during the entire time of simulation (Figures 11C, 12C). Remarkably, there were few fluctuations in the SASA values of compound 4 for very short time, but the greatest SASA value exhibited was 300 Å^2^ only. These values indicate that a substantial portion of compounds 3 and 4 are fitted relatively deeply in the binding pocket and have the least exposure to the solvent.

As illustrated in Figure 5B, there were three H-bond interactions, namely, Gln222, Ser226, and Lys555, of compound 4/PlmIX XP-docking pose. The evaluation of MD trajectory of control ligand 49C (Figures 9A, B) did not display any significant H-bond interactions with PlmIX, where the only H-bonds were formed with ASP-379, HIS-609, and THR-610 for less than 25% of the simulation time. A timeline representation of the interactions and contacts (H-bonds, Hydrophobic, Ionic, Water bridges) is represented in (Figure 9D). However, the MD trajectory assessment of control I0L (Figures 10A, B) demonstrated only two H-bond interactions with Gln222 and SER-226 (approximately 35% of MD trajectory). A timeline representation of the interactions and contacts (H-bonds, Hydrophobic, Ionic, Water bridges) is represented in (Figure 10D).

The evaluation of MD trajectory for compound 3 (Figures 11A, B) revealed that the complex 3/PlmIX established two hydrogen bonding interactions with Arg585 (77% of MD trajectory) and Leu552 (83% of MD trajectory). Furthermore, the complex also established one π–cation interaction with Arg585 (75% of MD trajectory). A timeline representation of the interactions and contacts (H-bonds, Hydrophobic, Ionic, Water bridges) is represented in (Figure 11D).

In comparison with the control ligands, the complex of compound 4/PlmIX (Figures 12A, B) displayed two H-bond interactions with Gln222 (48% of MD trajectory) and Ser226 (62% of MD trajectory). In addition to these bonds, a few other interactions were also seen within the MD trajectory, including one π–π stacking interaction with Phe500 (37% of MD trajectory) and three π–cation interactions with Lys555 (31% of MD trajectory), Arg335 (39% of MD trajectory), and Arg335 (47% of MD trajectory). A timeline representation of the interactions and contacts (H-bonds, Hydrophobic, Ionic, Water bridges) is represented in (Figure 12D).

## 4 Conclusion

In summary, our study aimed to identify novel and potent inhibitors of the PlmIX protein, with the ultimate goal of addressing antimalarial resistance. The initial screening of the OTAVA general fragment library using high-throughput virtual screening (HTVS) and molecular docking techniques yielded promising candidates for further investigation. We focused on fragments exhibiting XP docking scores ≤ −3 kcal/mol, which were then subjected to ligand breeding and subsequent molecular docking studies. From a pool of approximately 14,078 potential ligands, further refinement narrowed down our selection to the top 450 ligands, which underwent binding free energy calculations using the Prime MM–GBSA approach. Rigorous screening and analysis led us to prioritize 20 compounds with XP docking values ranging from −5.442 to −6.195 kcal/mol. Our analysis revealed critical interactions, including hydrogen bonds, π–cation bonds, and salt bridges, formed with amino acids such as Gln222, Ser226, Lys378, Asp379, Ser549, Arg554, Kys555, and Arg585 within the PlmIX-binding site. To validate the reliability of our findings, we conducted extensive molecular dynamics (MD) simulations, comparing the behavior of the top complexes with the Apoprotein and control ligands. These simulations demonstrated that compounds **3** and **4** exhibited greater interaction profiles than the control ligands, suggesting their potential as effective inhibitors of PlmIX. Although the results of our MD simulations are promising, we acknowledge that these are preliminary findings that require further validation through in-depth experimental studies. Our next step is to synthesize compounds **3** and **4**, along with their analogs, for comprehensive *in vitro* and *in vivo* testing. This experimental validation will provide crucial insights into the efficacy, safety, and clinical potential of these compounds as antimalarial agents. In conclusion, our research contributes to the ongoing efforts in antimalarial drug discovery, offering new insights and potential therapeutic candidates to combat antimalarial resistance. We are optimistic about the future impact of our work and remain committed to advancing the field through rigorous experimentation and validation.

## Data Availability

The original contributions presented in the study are included in the article/Supplementary Material; further inquiries can be directed to the corresponding author.
