# Supplementary figures and images for: Fragment-based virtual screening identifies novel leads against Plasmepsin IX (PlmIX) of Plasmodium falciparum: Homology modeling, molecular docking, and simulation approaches
Source: Front Pharmacol. 2024 May 23;15:1387629. doi: 10.3389/fphar.2024.1387629 (PMC11153788; doi:10.3389/fphar.2024.1387629)

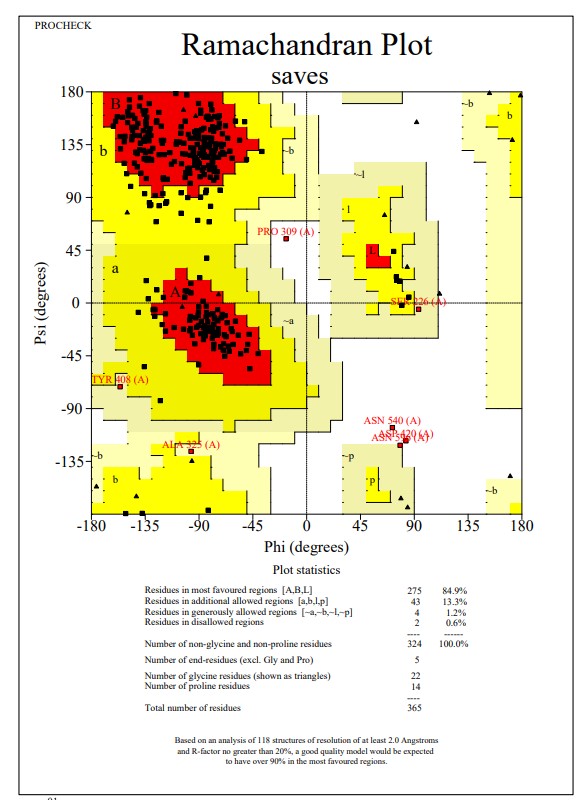

Supplement: Supplementary file 1 [file DataSheet1.ZIP › plm9 suplementery files/ramachandaran plot statistics 1.jpg]
